# Supplementary material for: Protection of hamsters challenged with SARS-CoV-2 after two doses of MVC-COV1901 vaccine followed by a single intranasal booster with nanoemulsion adjuvanted S-2P vaccine
Source: Sci Rep. 2022 Jul 5;12:11369. doi: 10.1038/s41598-022-15238-y (PMC9255510; doi:10.1038/s41598-022-15238-y)
Supplement: Supplementary file 1 — Supplementary Information. [file 41598_2022_15238_MOESM1_ESM.docx]

**Supplementary Information for**

**Protection of Hamsters Challenged with SARS-CoV-2 after Two Doses of MVC-COV1901 Vaccine Followed by a Single Intranasal Booster with Nanoemulsion Adjuvanted S-2P Vaccine**

Yi-Jiun Lin^1^, Meei-Yun Lin^1^, Ya-Shan Chuang^1^, Luke Tzu-Chi Liu^1^, Tsun-Yung Kuo^2^, Charles Chen^1,3^, Shyamala Ganesan^4^, ^ǂ^Ali Fattom^4^, ^*^Vira Bitko^4^, and ^*^Chia-En Lien^1,5^

^1^ Medigen Vaccine Biologics Corporation, Taipei City, Taiwan

^2^ Department of Biotechnology and Animal Science, National Ilan University, Yilan County, Taiwan

^3^ Temple University, Philadelphia, PA 19122, USA

^4^ BlueWillow Biologics, Ann Arbor, MI 48105 USA

^5^ Institute of Public Health, National Yang-Ming Chiao Tung University, Taipei City, Taiwan

ǂ Current affiliation: Avicenna Biologics Consultant, LLC, Ann Arbor, MI 48105

*Corresponding authors: Vira Bitko: vira.bitko@bluewillow.com; Chia-En Lien: allenlien@medigenvac.com


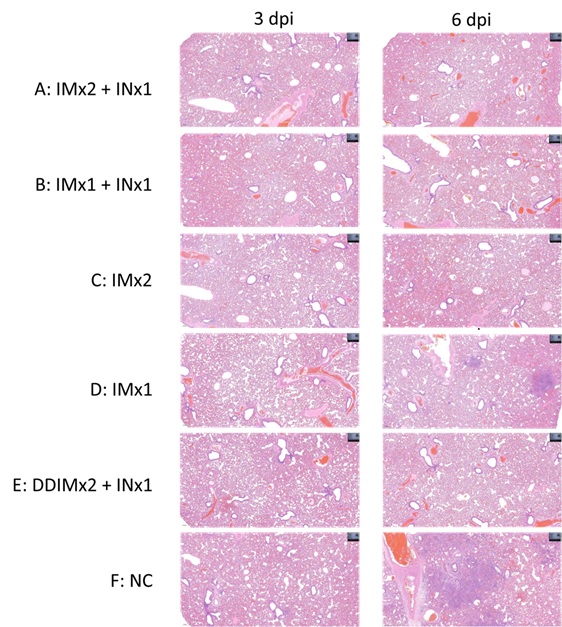


Figure S1. Representative histopathology sections of infected hamsters from Groups A to F at 3 d.p.i. or 6 d.p.i. The left lungs of hamsters were isolated and fixed in 4% paraformaldehyde for one week, sectioned and stained with Hematoxylin and Eosin for visualization.
